# Label-free quantification of early virus-induced cellular responses by digital holographic tomography

**DOI:** 10.1128/msphere.00304-26

**Published:** 2026-08-04

**Authors:** Agata Kublicka, Igor Buzalewicz, Dominika Skrzela, Leszek Moniakowski, Devon K. S. Fuller, Aleksandra Chwirot, Maja Marynowska, Grzegorz Chodaczek, Barbara Bażanów, Anna Karolina Matczuk

**Affiliations:** 1 Department of Pathology, Division of Microbiology, Faculty of Veterinary Medicine, Wroclaw University of Environmental and Life Sciences 686370, Wroclaw, Poland; 2 Bio-Quanty Group, Department of Biomedical Engineering, Wroclaw University of Science and Technology, Wroclaw, Poland; 3 Department of Biochemistry and Molecular Biology, Faculty of Veterinary Medicine, Wroclaw University of Environmental and Life Sciences 56641, Wroclaw, Poland; 4 Immunotherapy Research Group, Łukasiewicz Research Network – PORT Polish Center For Technology Development, Wroclaw, Poland; Helmholtz-Zentrum Dresden-Rossendorf, Görlitz, Germany

**Keywords:** digital holotomography (DHT), refractive index (RI), early virus-induced cellular responses, viral entry, equine viruses, EAV, EHV-1, ERBV, virus imaging

## Abstract

Early virus-induced cellular responses are a fundamental diagnostic feature of viral infections; however, early virus-host interactions, particularly during viral entry, remain difficult to study in living cells because many commonly used markers and antibodies require fixation or can interfere with cell viability. Although virus-induced cellular responses constitute a fundamental diagnostic feature of viral infections, their detection by microscopy typically relies on later-stage effects. Microscopic observation, therefore, serves as a complementary approach to molecular techniques, but the time required for visible changes to develop limits its utility for rapid assessment. Here, we propose a label-free digital holotomography (DHT) approach for the detection of early virus-induced cellular responses during the first hours of infection, including equine arteritis virus (EAV), equine herpesvirus 1 (EHV-1), and equine rhinitis B virus (ERBV). Using this method, we show that as early as 1–2 h post-infection, holotomography is capable of capturing virus-induced alterations in cellular density based only on changes in the refractive index (RI), which reflects local variations in cellular mass density and biochemical composition. In addition to global RI changes, we focused on subcellular alterations occurring at the early stages of infection, including modifications in lipid droplet-like structures and nucleoli. Our results reveal that these changes become detectable rapidly and can be captured with high sensitivity, displaying both cell type- and virus-dependent patterns. Our findings suggest that DHT may provide a rapid, label-free, and quantitative platform for detecting virus-induced subcellular changes as early as 1–2 h post-infection.

**IMPORTANCE:**

Digital holotomography (DHT) facilitates the early, label-free detection of infection and serves as a complementary diagnostic tool, allowing the rapid initiation of treatment. This method is universal and can be broadly applied to the investigation of primary host interactions, including general cellular and biomolecular optical density changes. These applications provide a strong foundation for viral entry inhibitor testing. Moreover, this approach highlights the critical importance of studying viral infections in natural host systems rather than relying solely on well-established experimental models. In this context, we employed equine lung cells (ELCs) as a biologically relevant and appropriate model to better reflect natural host-virus interactions. These findings should be interpreted in the context of an exploratory imaging data set and will require confirmation in replicate-based and longitudinal single-cell studies.

## INTRODUCTION

The isolation of viruses in cell culture remains a core method in virology. Although molecular assays such as qPCR are more sensitive for detecting viral nucleic acids, cell-based systems remain essential for studying early virus-induced cellular responses, virus neutralization, and antiviral activity. In practice, the analysis of cell infection events still relies heavily on microscopy-based approaches, most often combined with cell fixation and fluorescent labeling of viral or cellular structures (1, 2,(3)).

This creates a major technical limitation. Many commonly used methods for tracking early stages of infection depend on dyes or immunostaining, including lipid dyes, nucleic acid stains, or antibody-based detection of viral proteins. Such approaches can introduce phototoxicity, perturb membrane dynamics or intracellular trafficking, and, in many cases, require fixation, which prevents the continuous observation of the same living cell over time (4).

In addition, not all viral components can be easily labeled without affecting infectivity, and even early viral gene expression usually becomes detectable only after a delay. As a result, the initial cellular responses to infection are difficult to monitor in live cells using conventional microscopy.

Another challenge is that the morphological consequences of infection at very early time points are often subtle. Standard light microscopy may fail to detect such changes, especially when they do not yet produce clear cytopathic effects or when the analysis depends on the observer's experience. For this reason, label-free imaging approaches that allow monitoring of live-infected cells may provide an advantage for studying early post-entry virus-induced cellular responses.

Infection encompasses a spectrum of morphological alterations, including cell rounding, syncytial aggregation and inclusion formation (5), cytoskeletal remodeling (6), lipid droplet-like structures mobilization (7), and apoptosis involving nuclear fragmentation and cellular shrinkage (8). These alterations are often virus family-specific and reproducible. In order to enable earlier detection and improve prevention strategies against viral pathogens, it is clear that there is a need to evaluate newer imaging approaches that are tailored to virology research (9).

During entry, viruses trigger a series of rapid cellular changes, including receptor clustering at the membrane, actin remodeling, clathrin- or caveolin-mediated uptake, macropinocytosis, and endosomal trafficking (10, 11). Methods used to study these early events include confocal microscopy with tracking; co-localization with compartment markers and fusion reporters; image flow cytometry (12); and biochemical and molecular assays, such as bioacetylation (13), CRISPR/siRNA knockout/knockdown (14), inhibitor panels (15), endosome fractionation (16), and qPCR (17). These methods can map attachment, uptake, trafficking, and fusion well before classical cytopathic effect (CPE) appears. However, a key limitation is that these assays are complex and time-consuming and often require fixed cells along with fluorescent dyes, specific antibodies, or both, which can introduce perturbations and artifacts. This motivates the use of complementary label-free approaches, for example, quantitative phase imaging and holotomography, for minimally invasive assays.

Digital holotomography (DHT) is a label-free, non-destructive, real-time imaging method that quantifies how cells interact with light (18). It combines digital holography with optical diffraction tomography principles, where a coherent beam passes through the specimen, the induced phase shifts are reconstructed from digital hologram recorded for multiple illumination angles, and a tomographic algorithm reconstructs a 3D map of the sample’s refractive index (RI). Because the RI correlates with biomolecular density, DHT yields quantitative readouts such as cell and organelle volume, dry mass and dry mass density, refractive-index variance (a proxy for subcellular heterogeneity), and surface and shape metrics—all without dyes, fixation, or phototoxic exposure (19–21). This technique enables the quantitative, real-time assessment of RI changes and sample volume alterations, which can result from physiological and pathological processes or external factors, such as early virus-induced host response. DHT has previously been used to quantify infection-associated changes in RI and cell volume in cells infected with vaccinia virus, rhinovirus, and herpesvirus (18). It is also a valuable tool for investigating the pathological characteristics of different types of cells, monitoring changes in cells after exposure to various agents (e.g., chemicals, microorganisms, and nanomaterials), and observing quantitative dynamics in cells and their organelles (22).

EAV is an enveloped, positive-sense RNA virus that belongs to the *Arteriviridae* family. It replicates in the cytoplasm within replication organelles built from remodeled intracellular membranes. This virus causes equine viral arteritis, a respiratory-reproductive disease in which some stallions become long-term shedders (23).

Herpesviruses (*Herpesviridae*) are large, enveloped dsDNA viruses that replicate in the nucleus through a regulated immediate-early/early/leaky-late/late cascade; equine alphaherpesvirus-1 (EHV-1) produces acute respiratory disease and abortion in mares and can lead to fatal equine herpesvirus myeloencephalopathy (24).

ERBV belongs to the *Picornaviridae* family, a non-enveloped cytoplasmic positive-sense ssRNA virus; virion production follows virus-induced organelle remodeling, and particles are released mainly by cell lysis, with autophagy-linked egress also proposed (25, 26). Clinically, ERBV causes acute respiratory disease in horses, and its incidence has been rising globally (27).

Routine diagnosis of equine viral infections relies primarily on nucleic acid detection, including RT-qPCR for EAV and ERBV and qPCR for EHV-1 because these methods provide rapid and sensitive detection in biological samples. In serology, virus neutralization remains the gold standard for EAV according to the World Organization for Animal Health (WOAH) Terrestrial Manual ( 28) and is also used as a reference method for EHV-1. Virus isolation still serves as a functional confirmatory approach, but it depends on cytopathic-effect readouts such as cell rounding, detachment, or plaque formation, which makes it relatively slow and observer-dependent.

**Fig 1:**
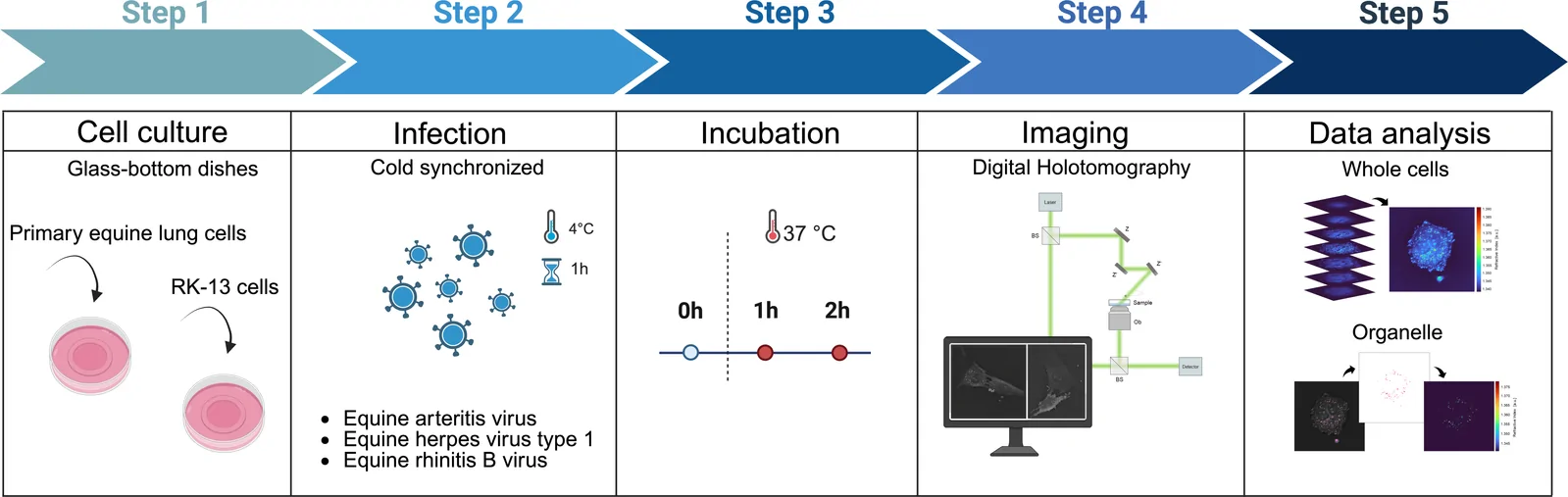
Schematic representation of the experimental workflow. Primary equine lung cells (ELCs) and the established rabbit kidney cell line (RK-13) were seeded onto Ibidi µ-slides in standard culture medium and incubated overnight at 37°C in a humidified atmosphere containing 5% CO₂ (step 1). Infection was synchronized by exposing the cells to EAV, EHV-1, or ERBV at a multiplicity of infection (MOI) of 50 for 1 h at 4°C (step 2). Following synchronization, the cells were transferred to 37°C and incubated for the indicated time points, 0, 1, and 2 hpi (step 3). Images were acquired using holographic tomography (step 4) with a 3D Cell Explorer microscope. For further analysis, images were reconstructed based on maximum refractive index (RI) values, and binary masks were generated (step 5).

At the same time, these equine viruses provide a useful experimental panel for methodological studies because they can be handled under BSL-2 conditions and represent distinct viral families with different biological properties. EAV, EHV-1, and ERBV, therefore, offer a relatively safe yet diverse model set for testing whether early virus-induced cellular responses can be detected before overt cytopathic effects become visible. In this context, they are well suited for the aim of this study: label-free quantification of early virus-induced cellular responses by digital holographic tomography across biologically distinct yet experimentally accessible virus-cell systems. The workflow is depicted in Fig. 1.

## MATERIALS AND METHODS

### Cells

The RK-13 cell line (ATCC CCL-37, Manassas, VA, USA), derived from rabbit kidney epithelial cells, was used in this study. The primary equine lung cells (ELCs) were derived from horse lung tissue as described earlier (29). Both cell lines were maintained and cultivated in Eagle’s minimum Essential medium (EMEM), supplemented with 10% fetal bovine serum (FBS; Certified Fetal Bovine Serum), 100 U/mL penicillin, 0.1 mg/mL streptomycin (penicillin-streptomycin solution), 100 mg/mL L-glutamine, and 1% non-essential amino acid solution, and incubated at 37°C in an atmosphere containing 5% CO_2_. All the above-listed media and supplements were acquired from Biological Industries (Northern Kibbutz Beit HaEmek, Israel).

### Viruses

Equine arteritis virus (EAV; Bucyrus reference strain), equine herpesvirus 1 (EHV-1; Valencia strain) (30), and equine rhinitis B virus (ERBV; Australian reference strain 1331/2/96) were propagated in RK-13 cells (propagated once to generate working stocks) and stored at −80°C. Virus stocks were titrated by the TCID_50_ method on the RK-13 cell line (31).

### Synchronized infections

Viruses were diluted in 20 mM HEPES-buffered DMEM without phenol red containing 0% FBS and penicillin-streptomycin. RK-13 and ELC cells were infected with the virus at an MOI of 50 and incubated on ice for 1 h. Cells were rinsed twice with cold phosphate-buffered saline (PBS) and overlaid with fresh medium supplemented with 2% FBS.

### Digital evaluation of the progression of CPE in light microscopy

ELCs and RK13 cells were seeded at a density of 3 × 10⁵ cells per well in 12-well transparent flat-bottom plates (Biologix, CA, USA) and subsequently infected with EAV, EHV, or ERBV at a multiplicity of infection of 50 following cold synchronization. Live-cell imaging was performed using the IncuCyte SX5 Live-Cell Analysis System (Sartorius BioAnalytical Instruments, MA, USA), maintained under standard cell culture conditions (37°C and 5% CO₂). Quantitative analyses were conducted with the IncuCyte 2022B Rev2 software using the Adherent Cell-by-Cell module. Phase-contrast images were acquired every hour over a 24-h period using a 10× objective, with nine fields captured per well at each time point. Image analyses were performed independently for each cell line to account for morphological differences. Time-lapse imaging was performed only by conventional light microscopy.

### Live label-free holographic tomography

For the DHT measurements, the ELCs and RK13 cells were seeded on 35-mm glass-bottom µ-dishes at a density of 3 × 10⁴ (Ibidi GmbH, Gräfelfing, Germany); 24 h after seeding, the cells were synchronously infected with EHV-1, EAV, or ERBV or were left uninfected. For DHT measurements, synchronized infected and mock-infected cells were analyzed at 0 h. Parallel dishes were then transferred to 37°C and incubated for 1 h or 2 h prior to imaging. Mock-infected controls were analyzed at 0 h and 2 h, whereas infected samples were analyzed at 0, 1, and 2 hpi.

A 3D Cell Explorer microscope (Nanolive, Ecublens, Switzerland), equipped with a Mach-Zehnder interferometer and a 60× dry objective lens (NA = 0.8), was used to capture three-dimensional refractive index (3D-RI) tomograms. The field of view (FOV) is 85 × 85 × 30 µm. The imaging system provided lateral and axial resolutions of 190 nm and 400 nm, respectively. A 520 nm scanning laser diode (0.2 mW/mm²) illuminated stationary samples from multiple angles.

To process the acquired digital holograms, STEVE software (v.1.6.3496, Nanolive, Ecublens, Switzerland) was utilized for deconvolution, visualization, and 3D-RI tomograms’ reconstruction. Each 3D-RI tomogram comprised 96 sequential 2D-RI tomograms. DHT measurements were acquired from separate samples at 0, 1, and 2 hpi for each virus. Between 12 and 30 3D-RI tomograms were obtained per infected and control sample, with a subset selected for further analysis.

DHT measurements were acquired from separate samples at 0, 1, and 2 h; therefore, the analysis represents population-level comparisons across early infection stages rather than longitudinal tracking of the same individual cells.

**Fig 2:**
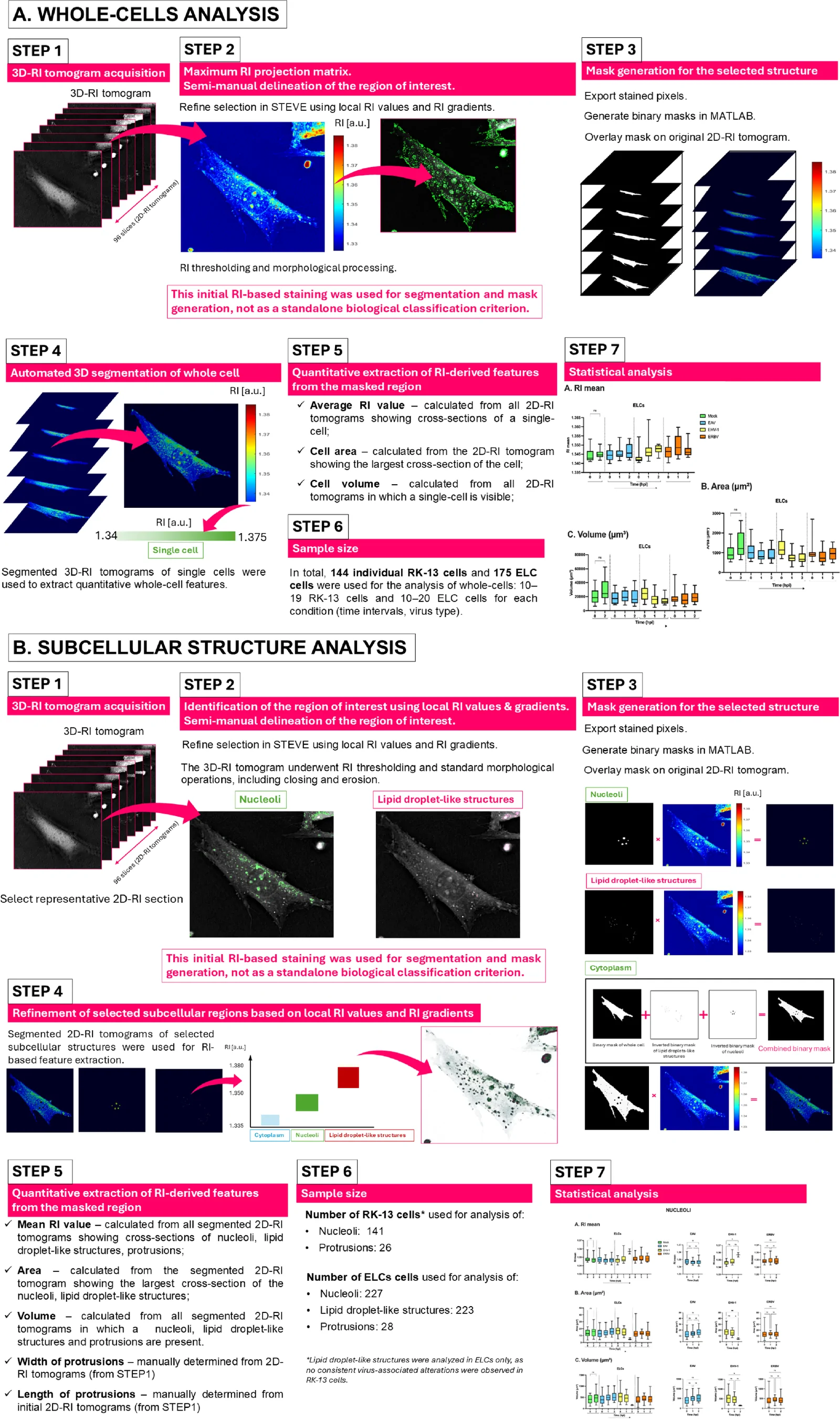
Schematic representation of the RI data-processing pipeline for whole cells and subcellular structures. (**A**) Whole-cell analysis pipeline. Step 1: 3D-RI tomograms were acquired. Step 2: maximum RI projection matrices were generated for each tomogram. Individual cells were then selected, and the approximate region of interest was semi-manually delineated in STEVE. Local RI values, RI gradients, thresholding, and morphological operations were used to refine segmentation and support binary mask generation. Step3: binary masks were generated in MATLAB and overlaid onto the original tomograms. Step4: Three-dimensional whole-cell segmentation was performed across sequential 2D-RI sections. Step5: Quantitative whole-cell features, including mean RI, cell area, cell volume, and dry mass, were extracted. Step 6: sample size is indicated. Step7: statistical analysis was performed across experimental conditions. (**B**) Subcellular structure analysis pipeline. Step1: 3D-RI tomograms were acquired and visualized in STEVE; representative 2D-RI sections were selected for further analysis. Step 2: cells with clearly discernible structures of interest were selected by visual inspection. The operator manually selected the 2D-RI section best representing the structure of interest and manually indicated the approximate region of interest. Step3: local RI values and RI gradients were then used in STEVE to refine the manually indicated region and isolate the corresponding pixels. Step4: the highlighted image was exported to MATLAB, where a binary mask was generated and overlaid onto the same original 2D-RI tomogram for quantitative extraction. Step5: quantitative features, including mean RI, standard deviation, area, volume, and protrusion-related measurements where applicable, were extracted from the masked region. Step6: sample size is indicated. Step7: statistical analysis was performed across time points and experimental conditions. RI-based staining was used as a semi-manual, image-guided segmentation aid for mask generation and quantitative extraction, rather than as an automatic biological classification step.

For subcellular structure analysis, the unit of analysis was a single cell. Within each analyzed cell, all clearly distinguishable structures of the selected type (e.g., nucleoli or lipid droplet-like structures) present in the chosen 2D-RI section were segmented, and quantitative parameters were calculated for the pooled segmented regions within that cell. In Fig. 2, we repeatedly show the same 2D-RI section for simplification and to illustrate the methodological workflow. Sample sizes varied depending on the cell type, virus, time point, and analysis type. For whole-cell analysis, 10-19 RK-13 and 10-20 ELC cells per condition were analyzed. For nucleolar analysis, 10-15 RK-13 and 10-29 ELC cells per condition were analyzed. For lipid droplet-like structures analysis, 10–15 RK-13 and 10–28 ELC cells per condition were analyzed. For protrusion analysis performed at 2 hpi, 7–11 RK-13 cells and 8–10 ELCs per condition were analyzed. Exact sample sizes for each condition are provided in Table S1.

MATLAB scripts (version R2020b, MathWorks, Natick, MA, USA) were implemented to extract RI values and obtain the 2D maps of maximal RI values from the examined volume. The data processing workflow involved thresholding and morphological operations to create binary masks for object segmentation.

### Whole-cell analysis RI

For whole-cell analysis, 3D-RI tomograms were imported into MATLAB, and a maximum RI projection matrix was generated for each 3D-RI tomogram to represent the cell’s largest cross-sectional area (Fig. 2A). Individual cells were then selected, and the approximate region of interest was semi-manually delineated. Local RI values, RI gradients, thresholding, and standard morphological operations, including closing and erosion, were used to refine segmentation and facilitate binary mask generation. The resulting binary mask was subsequently applied across all sequential 2D-RI tomograms (slices) constituting the 3D-RI tomogram, enabling three-dimensional segmentation of the selected whole cell.

Quantitative measurements included mean whole-cell RI, cell area, volume, and dry mass. Cell volume was calculated from the sum of segmented voxels across all 96 2D-RI sections using the voxel dimensions provided for tomogram reconstruction (“Quantitative analysis of 3D refractive index maps,” Nanolive, Switzerland). In total, 486 3D-RI tomograms were analyzed, corresponding to 46,656 individual 2D-RI slices. Dry mass was determined from RI measurements using a linear calibration model (32, 33).

Dry mass was included as an additional whole-cell parameter because it integrates RI-derived optical information with cell volume and therefore provides a complementary descriptor of infection-associated cellular changes. The dry mass was calculated based on the difference between the average RI of the analyzed organelle or cell (RIs) and the average RI of the surrounding medium (RI_medium), multiplied by the volume (V) and the refractive index increment (α). The refractive index increment (α), representing the proportionality constant between RI and concentration, was assumed to be 0.19 mL/g for all calculations.

### Single-cell variability analysis of whole-cell parameters

To characterize cell-to-cell heterogeneity in the DHT whole-cell data set, additional single-cell analyses were performed for ELCs and RK-13 cells. For each condition and time point, individual-cell values of cell area, cell volume, mean RI, and dry mass were visualized as single-cell distribution plots. To assess relative changes, baseline-normalized values were also calculated by dividing each single-cell measurement by the median value of the corresponding 0 h group within the same virus/cell-type combination.

To quantify relative dispersion, the coefficient of variation (CV = SD/mean), CV (%), and IQR-to-median ratio (IQR/median) were calculated for each parameter and group. These analyses were used to compare the relative stability of RI-based and morphometric whole-cell readouts across the studied conditions.

### Analysis of cell structures RI

For subcellular structure analysis, a separate RI-data processing pipeline was applied (Fig. 2B). Numerically reconstructed 3D-RI tomograms were visualized in STEVE software (v.1.6.3496, Nanolive, Ecublens, Switzerland). Each 2D-RI tomogram contains local RI values corresponding to distinct intracellular regions. The cells were first visually screened, and only cells with clearly discernible structures of interest were retained for further analysis. The operator then manually selected the 2D-RI section that best represented the structure of interest and manually indicated the approximate region of interest. Local RI values together with RI gradients were subsequently used within STEVE to refine this manually indicated area and isolate the corresponding pixels. Thus, RI-based staining was used as a semi-manual, image-guided segmentation aid for mask generation rather than as an automatic biological classification step. No fully automated classifier or independent cross-validation of segmentation was applied in the present study.

The highlighted image was then exported to MATLAB (version R2024b, MathWorks, USA), where a binary mask was generated by contrast inversion and overlaid onto the same original 2D-RI tomogram used for digital staining. Quantitative measurements were extracted exclusively from the masked region, and the mean RI and standard deviation were calculated using built-in MATLAB functions. In total, 453 3D-RI tomograms were processed, corresponding to 43,488 individual 2D-RI sections.

### Statistical analysis

All statistical analyses were performed using GraphPad Prism (version 10.6.1). Data normality was assessed using the Shapiro-Wilk test. For comparisons between mock-infected cells at 0 and 2 h post-infection (hpi), statistical significance was evaluated using an unpaired Student’s *t*-test when data followed a normal distribution with equal variances. Equality of variances was assessed, and in cases where this assumption was not met, Welch’s *t*-test was applied. When data did not follow a normal distribution, the nonparametric Mann-Whitney test was used. For the analyses of infection with different viruses over the time course (0, 1, and 2 hpi), comparisons across multiple groups were performed using one-way ANOVA when parametric assumptions were met. When significant differences were detected, post hoc multiple comparisons were conducted using Dunnett’s test (vs. control). When assumptions of normality were not satisfied, the nonparametric Kruskal-Wallis test was applied, followed by Dunn’s multiple comparisons test. All analyses were performed on independent (unpaired) samples. Data are presented as mean ± SD. A significance threshold of α = 0.05 was used.

### Immunostaining

F-actin staining was performed on subconfluent RK-13 and ELC cells infected with EAV, EHV, and ERBV. A cold-synchronized infection was conducted, and the cells were fixed with 4% paraformaldehyde for 12 min at different time points during early infection (0, 1, and 2 hpi). Following fixation, the cells were washed three times with PBS for 10 min and then incubated with Phalloidin iFluor 549 (Abcam Cambridge, UK) for 40 min at room temperature. After incubation, the cells were extensively washed three times with PBS and mounted using a DAPI mounting medium (Abcam, Cambridge, UK).

To visualize EAV particles on or within ELCS and RK-13 cells, a similar experiment was performed. Cells were synchronously infected with EAV at an MOI of 50, while mock-infected controls were inoculated with medium from non-infected RK-13 cells. After washing, the cells were incubated for 0, 1, or 2 h p.i. and subjected to an immunofluorescence assay as described earlier (34). Briefly, cells were then fixed, permeabilized, and blocked with 3% BSA (Sigma-Aldrich, Poland) for 1 h at room temperature. This was followed by incubation with mouse anti-N antibody (VMRD, Pullman, WA, USA), after which the secondary antibody, goat anti-mouse IgG H&L Alexa Fluor 488, was applied at a dilution of 1:800 (Abcam, Cambridge, UK) simultaneously with phalloidin iFluor 549 (Abcam, Cambridge, UK) for F-actin staining at a dilution of 1:1,000 for 40 min at room temperature. After the immunostaining process, cells were washed three times with PBS. All stained samples were subsequently examined using a confocal microscope.

### Confocal microscope imaging

Images were captured using a Zeiss Cell Observer SD confocal microscope (Zeiss, Oberkochen, Germany) equipped with an Andor iXon3 885 EMCCD camera and a 63× oil-immersion objective (pixel size 0.106 µm). Images were acquired sequentially using the 405 and 561 nm lasers and together with a quadruple dichroic mirror (405/488/561/640). Fluorescence emission was collected using a 450/50 filter for DAPI, a 520/35 filter for Alexa Fluor 488, and a 600/52 filter for Alexa Fluor 594. Image processing was performed using Fiji software.

## RESULTS

### Time-lapse recordings following infection of different cell lines with various viruses revealed distinct kinetics ofvirus-induced changes in development

To visualize the development of viral infection and determine the earliest visible onset, we performed automated live-cell imaging for 24 h during infection using automated light microscopy. After cold synchronized infection with EAV, EHV, or ERBV at an MOI of 50, multiple images of the cells were taken in the automated camera system installed in the cell culture incubator. The resulting image sequences (movies) are provided in reference 35.

Manual analysis of the recordings showed that in ELCs, the earliest detectable signs of CPE appeared at approximately 12 hpi for EAV, with a well-defined phenotype observed by 18 hpi. For EHV-1, initial CPE became evident at 5 hpi and developed into a clearly defined phenotype by 13 hpi. In contrast, ERBV induced visible cellular changes as early as 3 hpi, reaching a pronounced effect by 5 hpi. Mock-infected ELC cells represent physiological cell divisions over time.

In the RK-13 cell line, CPE progression followed a similar but slightly delayed pattern. For EAV, the first signs appeared around 12 hpi, with clearly defined changes evident by 22 hpi. EHV-1 infection produced early alterations at 7 hpi, with well-developed CPE at 13 hpi. ERBV infection resulted in an early response at 4 hpi, reaching peak visibility around 13 hpi. Mock-infected RK-13 cells represent physiological cell divisions over time.

These observations highlight the distinct kinetics of virus-induced morphological changes across different cell types, with ERBV showing the most rapid onset of infection, followed by EHV-1 and EAV.

For the automated assessment of CPE development, two features were quantified (Fig. S1) using the IncuCyte 2022B Rev2 software: mean object count and mean eccentricity. Mean object count represents the number of identified adherent cells, while mean eccentricity describes the degree to which cell shape deviates from a perfect circle.

### Early detection of virus-induced cellular alterations using the digital holographic tomography (DHT) method

**Fig 3:**
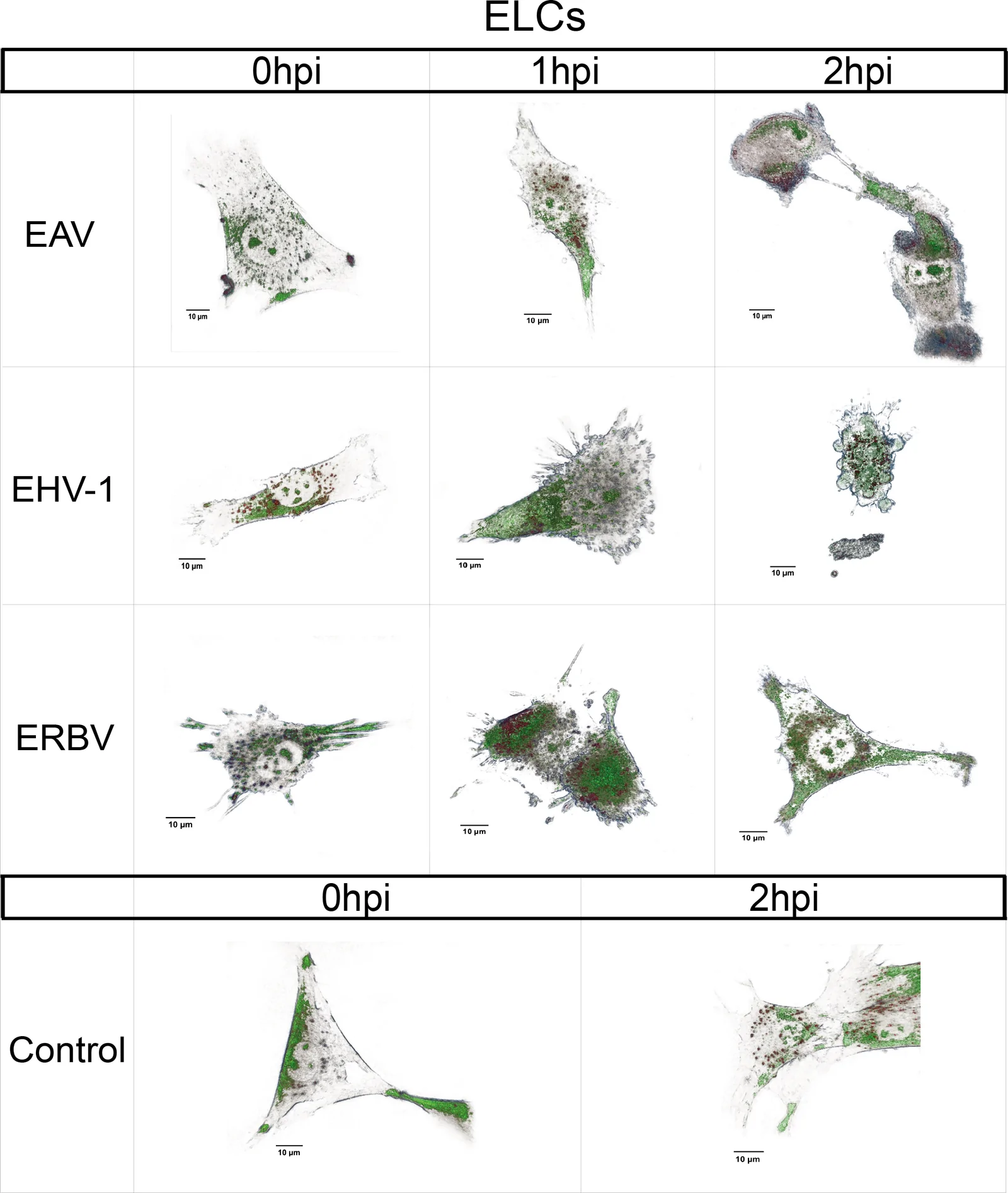
Representative morphology of ELCs infected with different equine respiratory viruses. The figures show representative images of ELCs infected with EAV, EHV-1, and ERBV, respectively. The primary ELCs exhibit irregular and elongated morphologies. At 0 hpi, compartmentation is visible in both infected and control cells, allowing clear visualization of organelle distribution. After 1 hpi, the first morphological alterations were observed, including the rearrangement of peripheral organelles toward the center of the cell, reflected by reduced visibility of organelles and corresponding changes in the distribution of pseudocolors. The most pronounced alterations occurred during EHV-1 infection, when the cells underwent changes in shape and shrinkage, resulting in a significant reduction in size and a loss of recognizable intracellular compartmentation. Representative images correspond to independent samples acquired at each time point; the same cells were not followed longitudinally.

**Fig 4:**
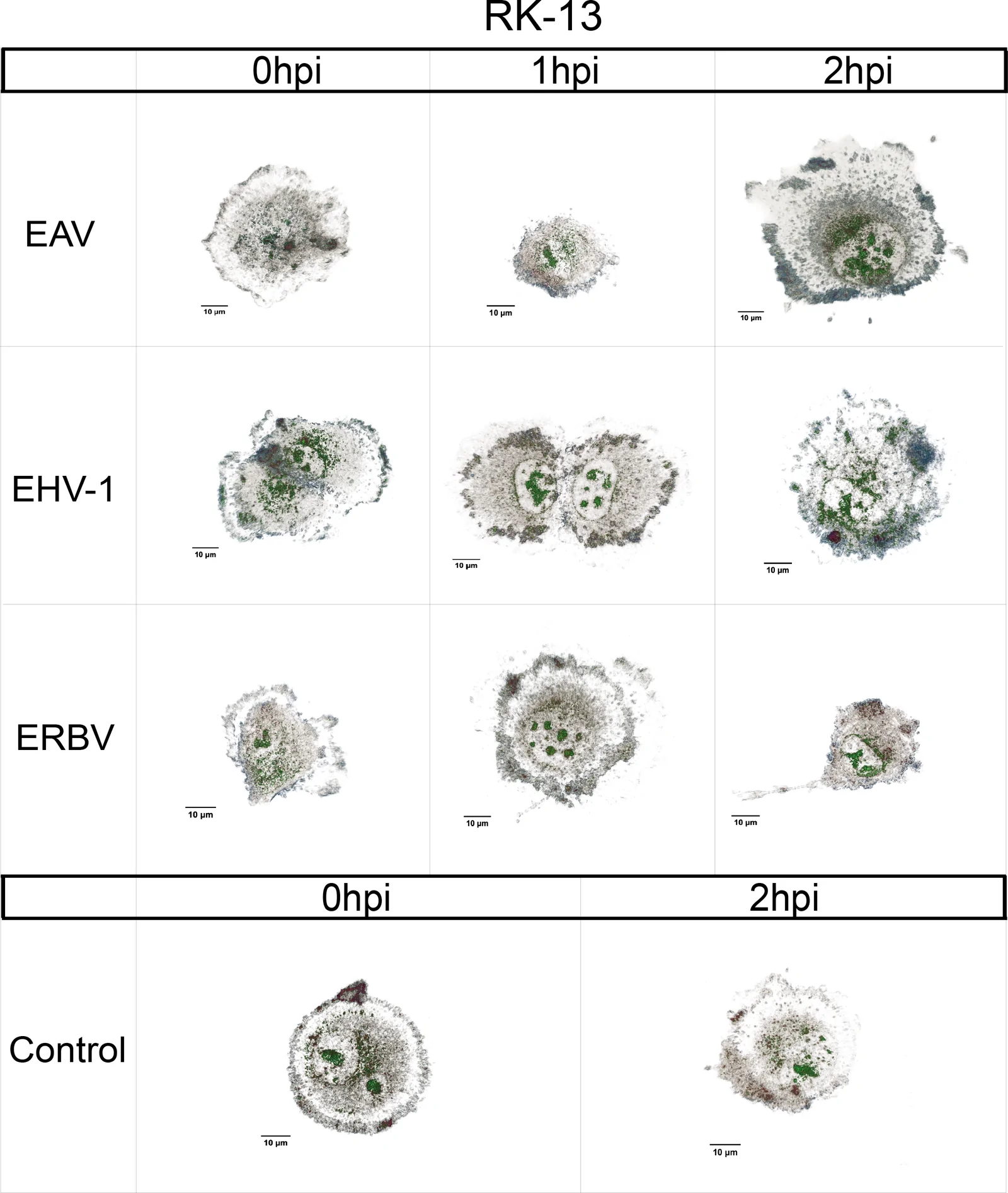
Representative morphology of RK-13 cells infected with different equine respiratory viruses. The panel shows established RK-13 epithelial-like cells, which are characterized by their regular, rounded morphology. In both uninfected and infected cells, a distinct cell outline was evident at 0 hpi. For EAV, the most pronounced changes were observed at 2 hpi, with alterations to the cell cortex presenting as a darker halo surrounding the cells. A similar effect was observed in EHV-infected cells, but this occurred earlier at 1 hpi, with intracellular compartmentation progressively deteriorating up to 2 hpi. For ERBV, the temporal pattern was similar to that observed for EHV-1, followed by cell shrinkage up to 2 hpi. Representative images correspond to independent samples acquired at each time point; the same cells were not followed longitudinally.

To visualize the early stages of viral infection, DHT was applied to cold-synchronized ELCs and RK-13 cells infected with either EAV, EHV-1, or ERBV at an MOI of 50. Between 12 and 30 3D-RI tomograms per condition were acquired at 0, 1, and 2 hpi and pseudocolored based on the RI to highlight structural changes (high RI, brown; intermediate, green; and low, blue). This pseudocoloring approach highlights density variations within the cells and enables clearer comparison of morphological changes across conditions. Representative images of all viruses at each time point during the infection are presented for ELCs in Fig. 3 and for RK-13 in Fig. 4.

In ELCs, we observed more severe changes compared with RK-13. The first infection-related changes appeared at 1 hpi, including changes in cell size, contraction, organelle clustering, and changes related to increased optical density toward the perinuclear region, with the most pronounced effects observed in EHV-1 and ERBV infections, and slightly weaker changes in EAV. By 2 hpi, the most significant remodeling of the cell membrane appears for EHV-1, and changes in cell morphology become difficult to define. In EAV-infected cells, the clarity of the internal cellular compartments tended to disappear, making the interior of the cell more uniform and dark, which was particularly evident 2 h after infection. In the case of ERBV, we observed changes in the shape and distribution of organelles over time, but to a lesser extent compared with EHV-1 and EAV.

RK-13 cells exhibited milder changes with a different morphological pattern. At 1 hpi, rearrangements were visible near the cell cortex just beneath the plasma membrane (common to all viral infections), but the clarity of cellular compartments remains clear. By 2 hpi, we observed further progression in all cases, visible as darker coloration across the cytoplasm.

Overall, DHT successfully detected early virus-induced morphological changes within the first 2 h of infection, differing in severity and pattern between cell types and viruses.

### Cellular alterations in refractive index patterns

**Fig 5:**
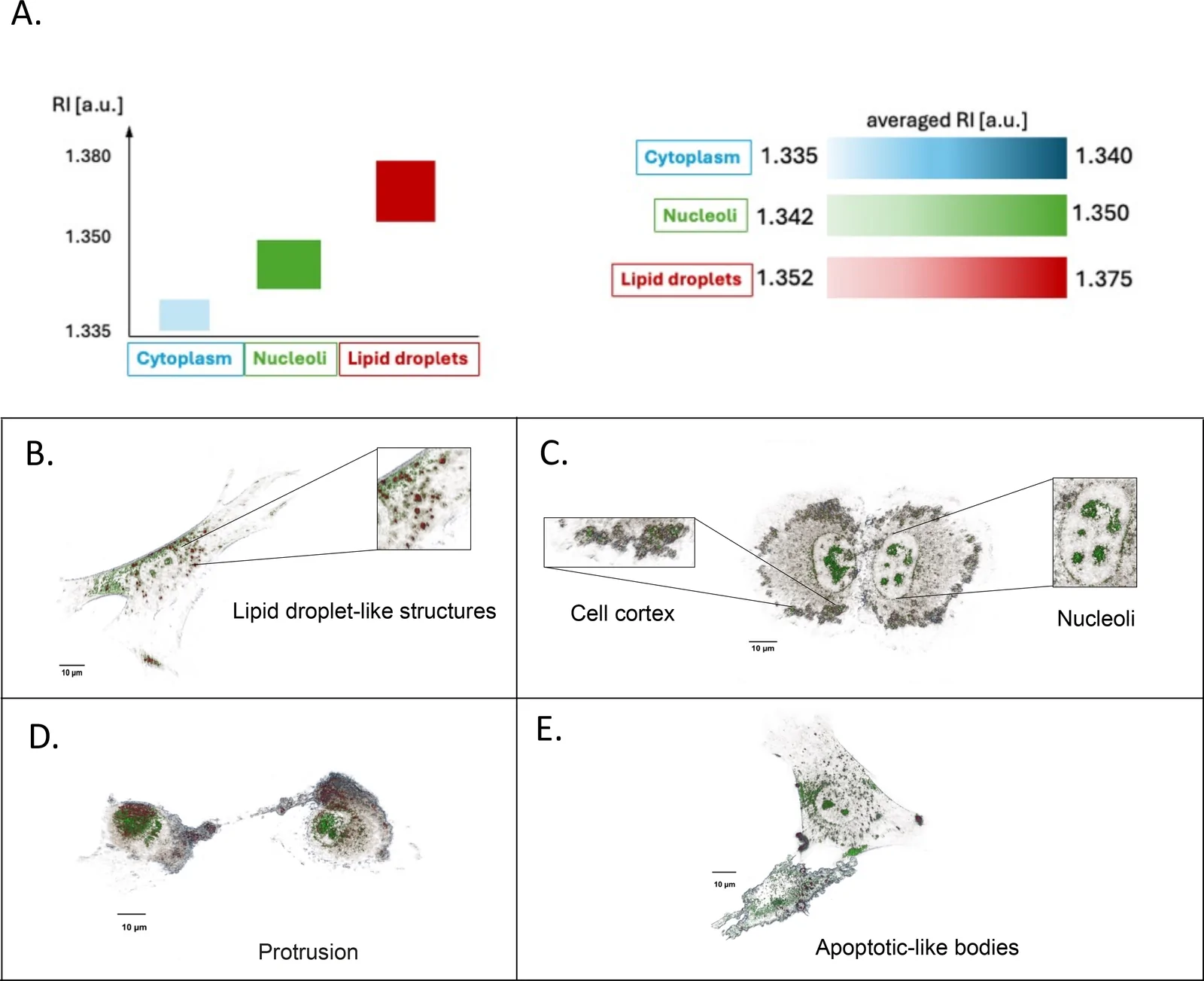
The most frequent early cellular changes observed in the infected cells. Digital holotomography images were displayed using pseudocolors that encode quantitative RI values. The pseudocolor scale represents increasing RI values, ranging from low to high, as indicated. The pseudocolor scale represents increasing RI values from low to high, as indicated. Low RI values (average RI 1.335–1.340 a.u.), shown in blue, correspond to the cytoplasm. Intermediate RI values (average RI 1.342–1.350 a.u.), displayed in green, correspond to nucleoli. High RI values (average RI 1.352–1.375 a.u.), shown in red, correspond to lipid droplet-like structures (**A**). The color bars shown in the schematic illustrate the mapping of pseudocolors used consistently across all images. Lipid droplet-like structures exhibited the highest RI values and therefore appeared red (**B**). In RK-13 cells, the inner cytoskeletal layer was particularly evident, manifesting as a darkly condensed area beneath the plasma membrane. This corresponds to high RI values, resulting in darker shading of the cell cortex (**C**, left). Medium RI values corresponded to nucleoli, which appeared as green foci (**C**, right). Protrusion formation was also observed (**D**), with their RI values approaching intermediate levels consistent with those of typical membranes and cytoplasm. Apoptotic-like cells were also identified (**E**). Overall, an increase in cellular RI results in darker coloration when these features appear, due to higher RI values.

DHT enabled the visualization of intracellular structures based on differences in the refractive index (RI). Figure 5 shows the reconstructed tomograms, where the most relevant characteristic changes observed during infection are highlighted. The color map scale, which reflects the RI values associated with various cellular patterns, is presented alongside the figure. The most frequent and recurring features were shown to vary for lipid droplet-like structures, nucleoli, the cell membrane, the cytoskeleton, and the formation of nanotubes, as well as for apoptosis-like changes. Redistribution of lipid droplets-like structures and nucleoli was a common feature across all viruses studied, whereas their rearrangement beneath the plasma membrane was observed exclusively in RK-13 cells. Protrusions were detected across all viral infections, while apoptotic-like changes were present only in selected cases. An increase in the RI reflects a higher optical density within the analyzed cellular region and can be interpreted as an increased local concentration of biomolecules.

### Time-dependent changes in cellular refractive index during infection

**Fig 6:**
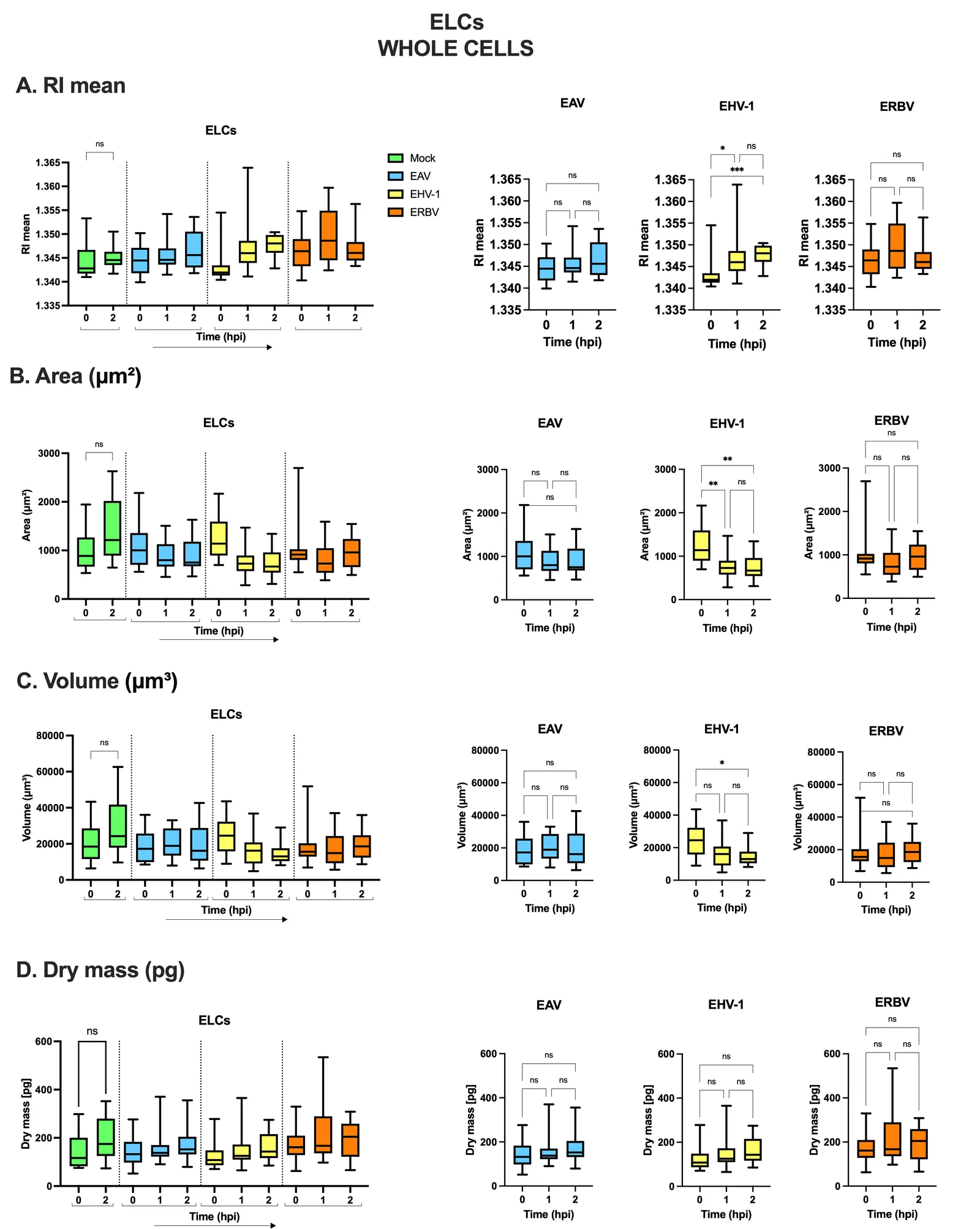
Early changes in the mean RI, area (µm²), volume (µm³), and dry mass during EAV, EHV-1, and ERBV infection in primary equine lung cells (*n* = 175). The figure presents a comparison of three parameters: mean RI, cell area, and cell volume in ELCs infected with different respiratory viruses: EAV (blue), EHV-1 (yellow), and ERBV (orange). Box plots represent the standard deviation, while bars inside the boxes indicate the mean values. Across the 0–2 hpi time course, EAV-infected ELCs exhibited only a slight increase in mean RI (**A**), which was not statistically significant. No significant changes were observed in cell area (**B**), volume (**C**), or dry mass (**D**). In contrast, EHV-1 infection led to a progressive and statistically significant increase in RI in primary ELCs between 0 and 1 hpi (*P = 0.0271) and between 0 and 2 hpi (***P = 0.0010). Conversely, cell area decreased significantly at both 1 hpi and 2 hpi compared with 0 h (**P = 0.0024 and **P = 0.0019, respectively), and the cell volume was also significantly reduced at 2 hpi (*P = 0.0291) relative to baseline. For ERBV-infected ELCs, RI values increased at 1 hpi and showed a declining trend at 2 hpi; however, these changes were not statistically significant. Likewise, no significant differences in cell area, volume, or dry mass were observed across any of the virus conditions. ns, not statistically significant.

**Fig 7:**
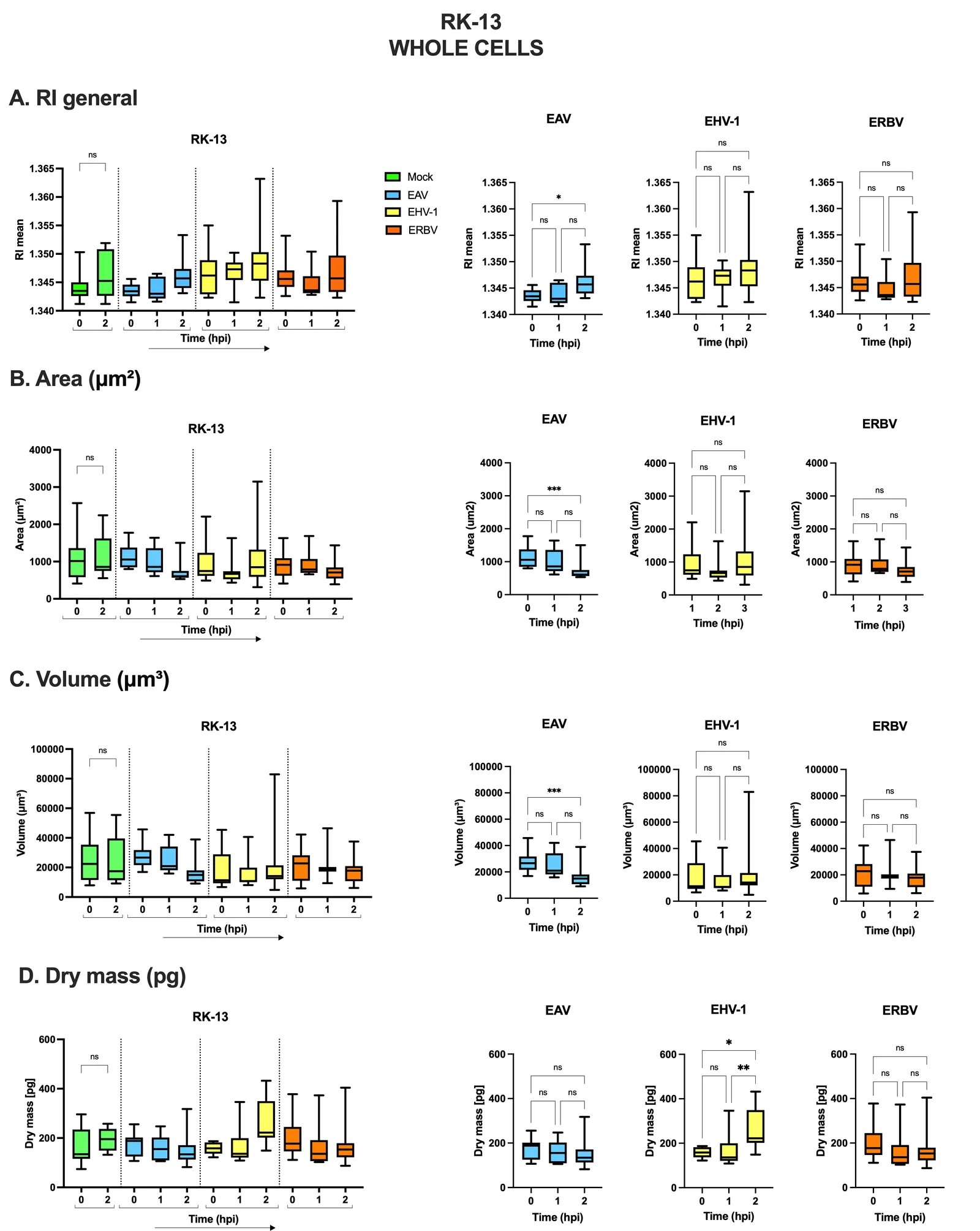
Early changes in the mean RI, area (µm²), volume (µm³), and dry mass during EAV, EHV-1, and ERBV infection in RK-13 cells (*n* = 145). The figure presents a comparison of three parameters: mean RI, cell area, and cell volume in RK-13 infected with different respiratory viruses: EAV (blue), EHV-1 (yellow), and ERBV (orange). Box plots represent the standard deviation, while bars inside the boxes indicate the mean values. For EAV-infected cells, a statistically significant increase in mean RI (**A**) was observed between 0 and 2 hpi (*P = 0.0197). In contrast, cell area (**B**) and volume (**C**) showed an overall decrease, with significantly lower values at 2 hpi (***P = 0.0005 and ***P = 0.0004, respectively). Dry mass (**D**) remained unchanged, with no statistically significant differences observed. In the case of EHV-1, all parameters exhibited an overall increasing trend, with a slight decrease at 1 hpi followed by a subsequent rise. Statistically significant differences were observed only for dry mass, between 0 and 2 hpi (*P = 0.0193) and between 1 and 2 hpi (**P = 0.0029). For ERBV, mean RI showed a slight decrease at 1 hpi, followed by an increase at 2 hpi. Cell area, volume, and dry mass remained largely stable throughout the time course. ns, not statistically significant.

General cellular alterations were assessed by quantifying the mean RI, area (µm²), defined as a surface area of the reconstructed 3D object, volume (µm³), and dry mass corresponding to the sum of segmented voxels of individual cells in ELCs (Fig. 6) and RK-13 cultures (Fig. 7). Single-cell segmentation was performed using manual cell masking, and the measurements were analyzed at the same time points as before (0, 1, and 2 hpi). Uninfected controls were included at 0 and 2 hpi to determine the potential physiological changes over time. No significant differences were observed between these time points in either uninfected cell line, confirming stable conditions and excluding potential technical impairments arising from washing steps, media replacement, or synchronization at the low temperature.

To further assess cell-to-cell heterogeneity within the DHT data set, we visualized individual-cell distributions for whole-cell area, volume, mean RI, and dry mass in ELCs and RK-13 cells and additionally normalized these parameters to the corresponding 0 h baseline within each virus/cell-type group. These analyses showed that morphometric parameters, particularly cell area and cell volume, displayed substantial heterogeneity at the single-cell level in both cell models. Dry mass showed a similarly broad distribution, whereas the mean RI exhibited comparatively tighter distributions across conditions. Quantitative variability analysis confirmed this observation, as mean RI showed markedly lower coefficients of variation and lower IQR-to-median ratios than area, volume, and dry mass in both ELCs and RK-13 cells. Thus, although population-level trends remained detectable in several infection conditions, the additional single-cell plots indicate that early virus-induced cellular responses are heterogeneous and that different DHT-derived parameters capture distinct aspects of this variability. The comparatively high variability of dry mass, despite the relative stability of mean RI, likely reflects its composite nature, as dry mass depends not only on RI-related optical properties but also on cell volume. Accordingly, the pronounced heterogeneity of cell volume appears to contribute substantially to dry mass variability. This pattern is summarized in Table 1, which shows that mean RI displayed markedly lower relative variability than area, volume, and dry mass in both cell models. The corresponding single-cell distribution plots, baseline-normalized analyses, and full variability metrics are provided in Fig. S2 to S5 and in reference 36.

**TABLE 1:** Relative variability of whole-cell parameters across analyzed conditions in ELCs and RK-13 cells^*a*^

Cell type	Parameter	Median CV (%)	Range (%)
ELCs	Area	40.3	32.9–50.0
ELCs	Volume	53.3	38.9–62.3
ELCs	Mean RI	0.27	0.18–0.62
ELCs	Dry mass	42.7	35.4–58.7
RK-13	Area	39.1	25.5–52.7
RK-13	Volume	52.3	31.2–66.6
RK-13	Mean RI	0.21	0.14–0.31
RK-13	Dry mass	37.4	24.6–50.7

^*a*^ For each condition and time point, variability of whole-cell area, volume, mean RI, and dry mass was quantified using the mean, standard deviation (SD), median, interquartile range (IQR), coefficient of variation (CV), CV (%), and IQR-to-median ratio. Mean RI showed markedly lower relative dispersion than cell area, cell volume, and dry mass in both cell models, suggesting that RI-based measurements may provide a comparatively more stable descriptor for population-level comparisons.

### Differences in nucleoli and lipid droplet-like structures RI over the time course of infection with different viruses

**Fig 8:**
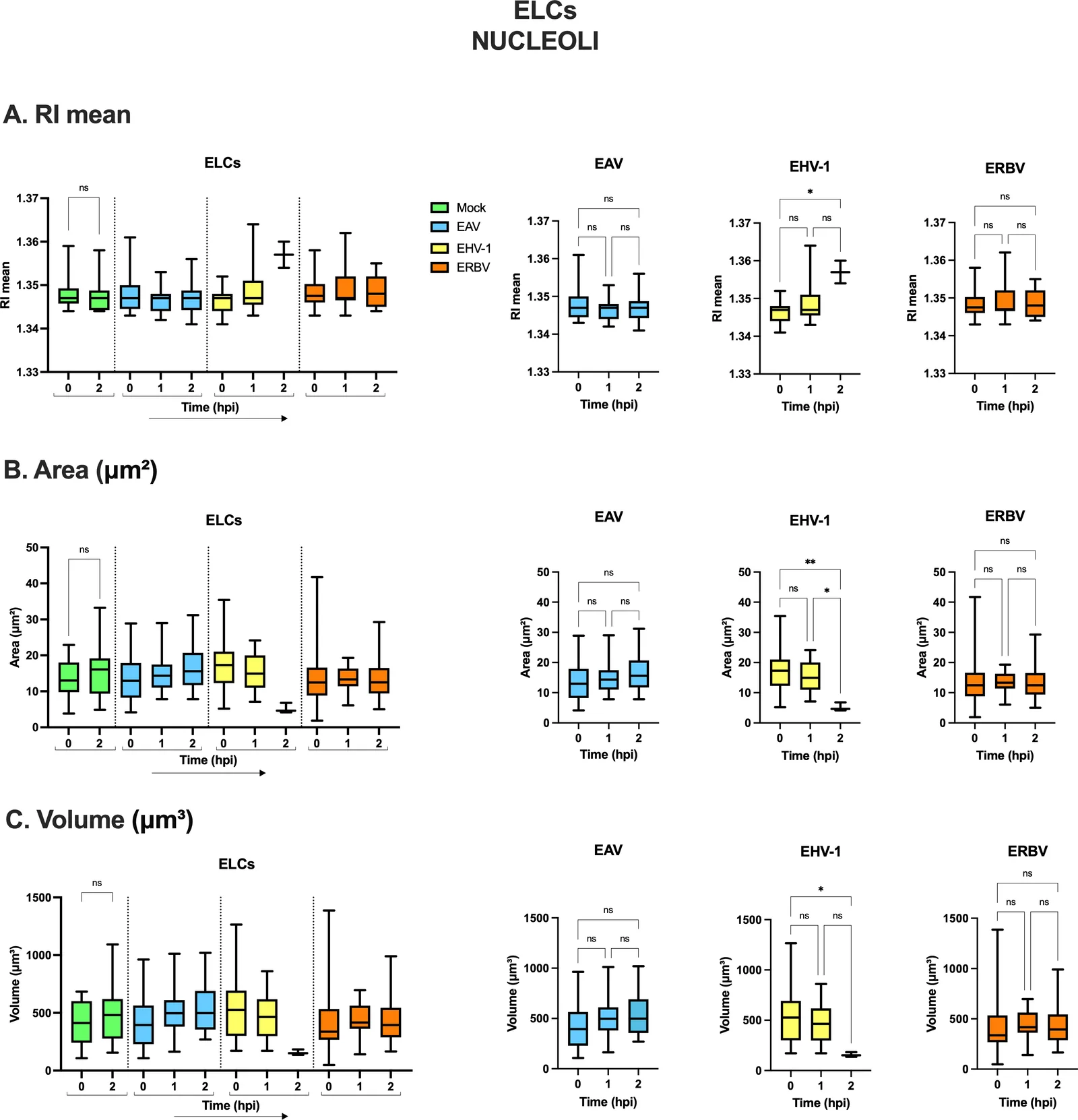
Early changes in the nucleoli mean RI, area (µm²), and volume (µm³) during EAV, EHV-1, and ERBV infection in primary equine lung cells (*n* = 227). In primary ELCs, nucleolar RI values (**A**) remained largely unchanged during EAV infection, whereas EHV-1 infection resulted in a statistically significant difference between 0 and 2 h post-infection (*P = 0.0201). No statistically significant changes in mean RI were observed for ERBV. Analysis of nucleolar area (**B**) and volume (**C**) revealed increasing trends during EAV infection, in contrast to EHV-1 infection, which was associated with a statistically significant decrease in nucleolar area between 0 and 2 h (**P = 0.0058) and between 1 and 2 h post-infection (*P = 0.0281). A significant decrease in nucleolar volume was also observed for EHV-1 between 0 and 2 h post-infection (*P = 0.0239). No clear trends or statistically significant differences in nucleolar area or volume were detected during ERBV infection. ns, not statistically significant.

**Fig 9:**
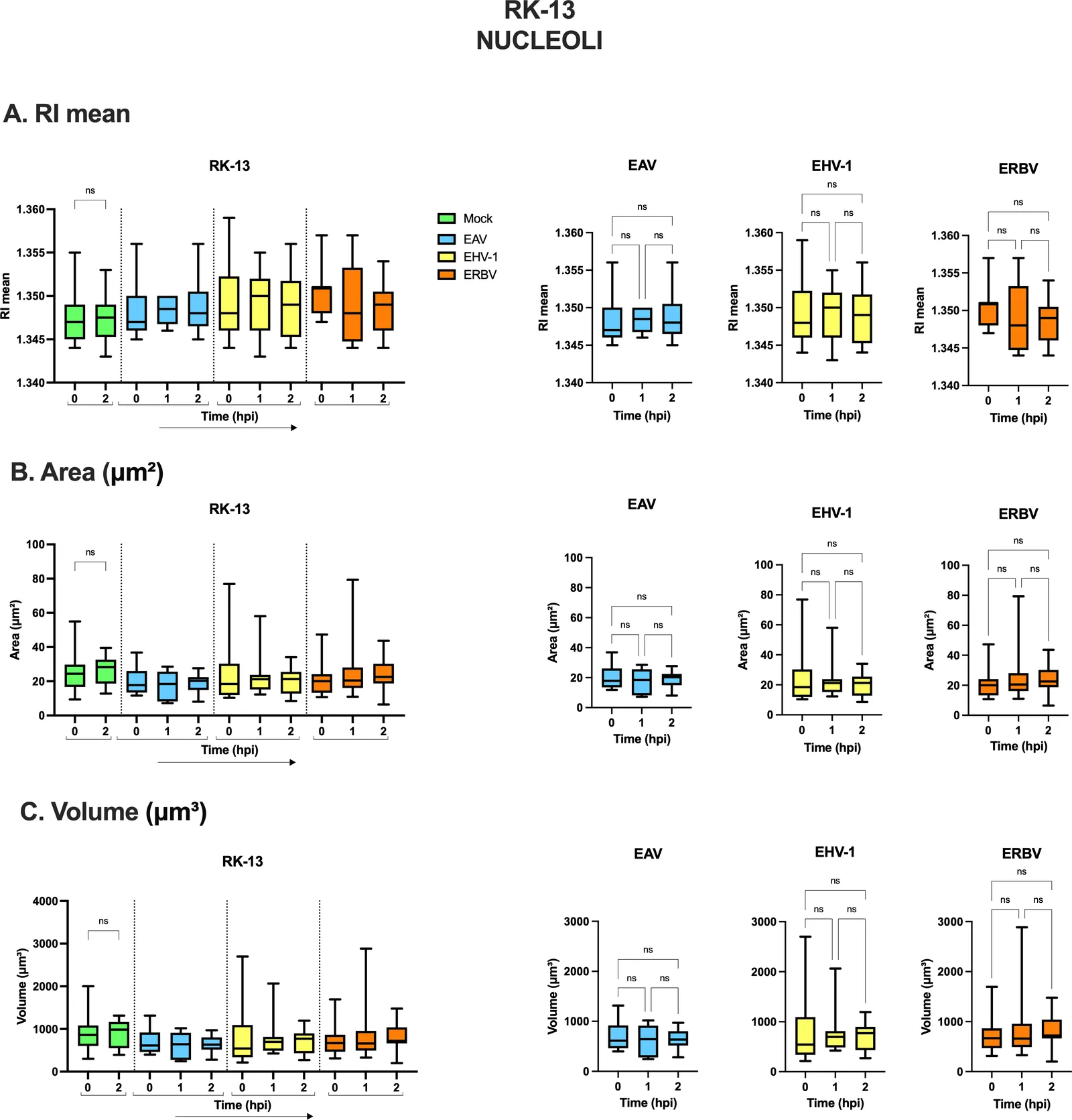
Early changes in the nucleoli mean RI, area (µm²), and volume (µm³) during EAV, EHV-1, and ERBV infection in RK-13 cells (*n* = 141). In RK-13 cells, no statistically significant differences were detected in the nucleoli refractive index (**A**), area (**B**), or volume (**C**) across the infection time course for any of the viruses studied. EAV and EHV-1 infections were characterized by an initial increasing trend in nucleolar RI up to 1 hpi, followed by a decreasing trend, whereas ERBV infection showed the opposite trend, with an initial decrease in RI followed by a gradual increase. Analysis of the nucleoli area and volume revealed a relatively stable size in EAV-infected cells. For EHV-1 and ERBV infection, a consistently decreasing trend was observed. Despite these observed trends, none of the changes have reached statistical significance. ns, not statistically significant.

To investigate local changes that may be virus-induced, we analyzed the RI, surface area, and volume of nucleoli and lipid droplet-like structures. In primary equine lung cells (Fig. 8), no statistically significant changes over the course of infection were observed for EAV or ERBV in any of the analyzed parameters (RI, surface area, or volume). In contrast, EHV-1 infection resulted in a statistically significant increase in nucleolar RI, accompanied by a significant decrease in both nucleolar volume and surface area within 2 hpi. These effects were observed exclusively for EHV-1 and only in primary cells, but not in the stable RK-13 cell line, in which no statistically significant differences were detected for these parameters (Fig. 9) across all virus families.

**Fig 10:**
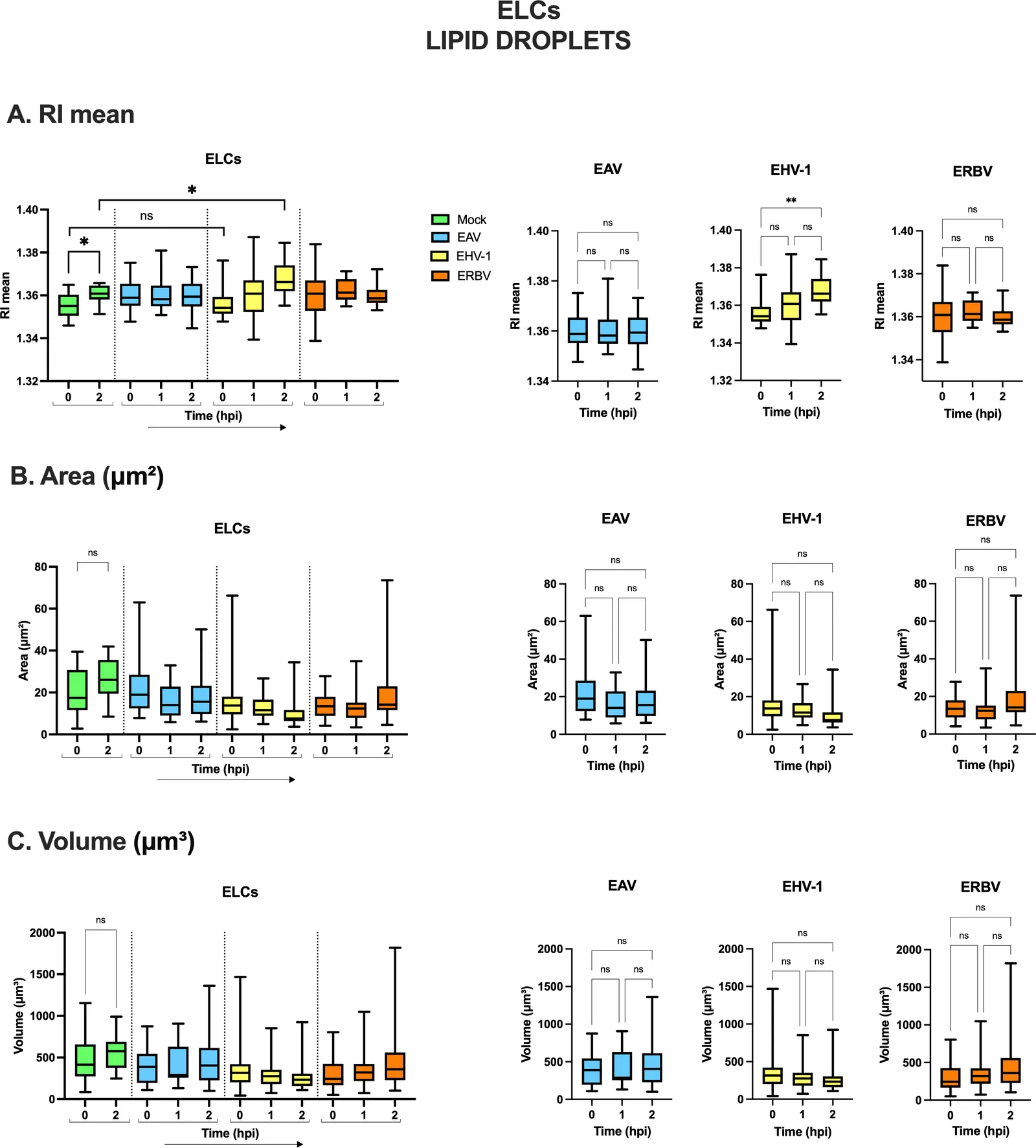
Early changes in lipid droplet-like structures’ mean refractive index, area (µm²), and volume (µm³) during EAV, EHV-1, and ERBV infection in ELC cells (*n* = 223). Quantification of RI, area, and volume was possible only for primary equine lung cells. Comparison of control cells between 0 and 2 h revealed a statistically significant increase in mean RI (*P = 0.0270) (**A**). An increasing trend in RI was observed in primary cells infected with EHV-1, with a statistically significant difference between 0 and 2 hpi (*P = 0.0037). However, there were no statistical differences between control and infected cells measured for EHV-1 at 0 hpi, while a statistically significant increase was observed when comparing control and infected cells after 2 hpi (*P = 0.0345). In contrast, EAV-infected cells indicated stable RI values over the infection period. ERBV infection was associated with a decreasing trend in RI that did not reach statistical significance. Analysis of the nucleoli area (**B**) revealed a slight initial decrease followed by an increase between 1 and 2 hpi for both EAV- and ERBV-infected cells, whereas a consistently decreasing trend was observed during EHV-1 infection. For nucleoli volume (**C**), increasing trends up to 2 hpi were observed for EAV and ERBV, while EHV-1 infection was associated with a decreasing trend. However, no statistically significant differences were detected for area or volume for any of the viruses. ns, not statistically significant.

Lipid droplet-like structures could be quantified only in the primary ELCs (Fig. 10). Statistically significant changes were observed exclusively for EHV-1 infection, with a significant increase detected between 0 and 2 hpi. Additionally, a statistically significant difference with an increasing trend was observed between control time points.

### Quantitative assessment of cell protrusions and intercellular connections induced upon virus infection demonstrated the feasibility of measuring these structures

Image analysis revealed the presence of intercellular linkages connecting infected ELCs and RK-13 cells at 2 hpi. In ELCs, representative images of tubular intercellular connections were identified for EAV, EHV-1, and ERBV infections. Quantitative analysis included measurements of mean RI, area, volume, and protrusion width and length. Due to the limited number of images in which tunneling could be distinguished in ELCs, only trends are presented for this cell type. Overall, EAV infection showed the highest values for most analyzed parameters, including RI, area, volume, and protrusion length. In contrast, the width of protrusions displayed the strongest increasing trend during EHV-1 infection (Fig. S6).

In RK-13 cells, a substantially higher number of tubular intercellular connections was detected, allowing quantitative and statistical analyses. No statistically significant differences were observed in the mean RI values among the viruses. However, statistically significant differences were detected between EAV- and EHV-1-infected cells in the volume, area, and width of tubular intercellular connections, with all three parameters being significantly higher during EAV infection, indicating larger and thicker protrusions. Additionally, protrusions formed during ERBV infection were significantly longer compared with those induced by EAV and EHV-1 (Fig. S6).

### Actin distribution in early infection time points

Given the limited data on actin organization during infection with these viruses, particularly in primary cell systems, we assessed actin staining patterns in infected and uninfected cells by confocal microscopy. Qualitative differences in actin distribution were observed at 2 hpi relative to control cells. However, these observations were not quantified, and no conclusions were drawn regarding the extent or mechanism of actin rearrangement. Such analysis would require dedicated quantitative approaches and was beyond the scope of the present study (Fig. S7 and S8).

### Visualization of EAV particles on cells

To assess the timing of early EAV infection events, viral particles were visualized by confocal microscopy in ELCs and RK-13 (Fig. S9), following synchronized infection with an inoculum MOI of 50. At 0 hpi, numerous EAV-positive particles were detected on the cell surface in both cell types. By 1 hpi, the number of detectable particles was strongly reduced, with most of the signal no longer visible. This observation is consistent with rapid virus internalization and uncoating, suggesting that the 1 hpi time point in EAV infection likely reflects a post-uncoating stage. Equivalent analyses could not be performed for EHV-1 and ERBV due to the lack of suitable antibodies for the visualization of viral particles.

## DISCUSSION

Cell culture infection models, particularly in primary cells, remain fundamental for studying virus-host interactions, as they enable direct observation of virus-induced cellular responses, including cytopathic effects (24, 37). In our study, we focused on three major equine respiratory viruses, EAV, EHV-1, and ERBV, which are clinically important pathogens causing respiratory disease in horses and belong to distinct viral families. Because all three viruses can be grown in the same cell type, we were able to conduct a direct comparative analysis of initial post-entry responses, as well as early cellular changes with new tools such as DHT.

Recent years have seen a growing number of studies applying DHT to analyze cells and cellular dynamics in a label-free, quantitative manner, including investigations of morphology, motility, and biophysical properties in cells (23, 38, 39).

To systematically compare infection dynamics, we performed time-lapse analyses using conventional light microscopy with the automated IncuCyte system. Because studies addressing the early stages of infection by equine viruses remain limited, especially in primary equine cells, we included this analysis to provide a broader comparative framework for interpreting the timing and progression of virus-induced cellular responses under conventional live-cell imaging conditions.

All experiments were conducted under cold-synchronized conditions at a high multiplicity of infection (MOI of 50), as commonly applied in virus entry studies to ensure near-uniform infection of the cell population and to minimize variability arising from asynchronous viral entry. This strategy enabled standardized comparisons and allowed us to determine the earliest time points at which virus-induced morphological changes become detectable by light microscopy.

Manual inspection of the recordings revealed clear, virus-specific differences in the kinetics of infection, consistent with known replication strategies of the respective viruses. ERBV induced rapid, cytocidal changes characterized by early cell rounding and damage, similar to those reported for some rhinoviruses (40), whereas EHV-1 showed intermediate kinetics associated with herpesvirus-specific cytoskeletal remodeling and cell fusion leading to syncytium formation (41, 42). In contrast, EAV exhibited the slowest progression, with pronounced cytopathic effects, including cell rounding and detachment, appearing only after 18–22 h post-infection, consistent with the delayed and initially subtle cellular alterations typical of arteriviruses and their caspase-dependent cell death pathways (30, 43).

Comparison with automated IncuCyte-based analysis showed strong concordance in the relative ranking of CPE kinetics (ERBV > EHV-1 > EAV), confirming the biological validity of manual observations.

However, while manual microscopy effectively captured overt morphological changes once they became visually apparent, automated quantification resolved earlier and more subtle infection-associated dynamics. In both cell lines, IncuCyte metrics refined manual findings by revealing early fluctuations in object count (reflecting the number of adherent cells, with decreases indicating CPE progression associated with cell detachment and death) and eccentricity (capturing infection-induced changes in cell shape; the rounder the cell becomes, the lower the eccentricity value). In ELCs, ERBV infection induced early changes in these metrics that preceded overt cell loss, whereas EAV and EHV-1 showed more gradual and uniform trends consistent with slower CPE development. In RK-13 cells, automated analysis further distinguished virus-specific processes, such as syncytium formation during EHV-1 infection and the biphasic response to ERBV, which were less apparent by visual inspection alone.

The generated bias may result from syncytium formation by herpesviruses, which reduces the number of detectable objects because fused cells acquire irregular shapes that are difficult for the segmentation algorithm to accurately identify. This effect is consistent with the distinct morphological responses of ELCs and RK-13 cells. ELCs, which display a fibroblastic and intrinsically irregular morphology, showed a progressive decrease in eccentricity during infection, reflecting their transition toward a more rounded cell shape. In contrast, RK-13 cells, which are epithelial-like and naturally more rounded, exhibited an increase in eccentricity driven by cell fusion and the formation of large, irregular syncytia (44, 45).

To our knowledge, these three clinically relevant equine viruses have not previously been compared side by side under identical conditions using this type of automated live-cell imaging approach.

The main aim of this work was to determine whether the earliest stages of viral infection can be measured and visualized by DHT without the need for cell labeling or fixation. Importantly, DHT allows quantitative three-dimensional imaging and direct measurement of the cellular refractive index, which reflects intracellular mass, density, and organization, thereby providing additional biophysical information on infection-associated structural and compositional changes beyond conventional morphological assessment (46, 47).

We hypothesized whether it is possible to identify early virus-specific changes based on the RI profiles of whole cells or particular organelles.

Whole-cell RI analyses were not uniform across infection models. In primary equine lung cells, the statistical increase in global RI was detected exclusively following EHV-1 infection, together with the concurrent reduction in the cell area and volume. Such early cellular compaction is in line with previous reports describing herpesvirus-induced cytoskeletal rearrangements and membrane-associated processes that precede overt morphological damage (48). A comparable relationship between RI and geometric parameters was observed in RK-13 cells; however, in this case, the effect was specific to EAV infection. Increases in RI were only detected under conditions where cell area and volume were simultaneously reduced, whereas mock-infected controls displayed neither RI shifts nor changes in these parameters. Importantly, the tight coupling between RI elevation and reductions in geometric parameters supports the notion that RI changes captured by DHT reflect genuine biophysical remodeling of the cell rather than nonspecific optical fluctuations.

The additional single-cell variability analysis provides an important methodological perspective on the interpretation of DHT-derived readouts. In both ELCs and RK-13 cells, morphometric parameters such as area and volume showed substantial relative dispersion, indicating that these readouts are strongly influenced by intrinsic cell-to-cell heterogeneity during early infection. Dry mass behaved similarly. By contrast, mean RI displayed markedly lower relative variability across the analyzed conditions. This suggests that RI-based measurements may provide a comparatively more robust descriptor for population-level comparisons of early infection-associated cellular changes, whereas morphometric and dry mass parameters capture a more heterogeneous dimension of the cellular response. An additional point of interest is that dry mass remained substantially more heterogeneous than mean RI despite the relative stability of RI across conditions. This is consistent with the composite nature of dry mass, which depends on both RI-related optical properties and cell volume. In this context, the markedly higher heterogeneity of volume appears to outweigh the stabilizing contribution of RI, resulting in substantial variability of dry mass at the single-cell level. Thus, dry mass should not be interpreted as a simple extension of RI, but rather as a compound readout integrating optical density and cell size. Accordingly, these readouts should be viewed as complementary rather than interchangeable: morphometric parameters are informative for describing heterogeneity and response amplitude, whereas the mean RI may be particularly useful as a stable quantitative parameter for early label-free phenotyping. At the same time, the distinct patterns observed between ELCs and RK-13 cells indicate that the biological context of early virus-cell interactions remains a major determinant of the measured DHT response.

Differences between the two cell types are likely influenced by variations in virus-cell entry mechanisms and host cell properties, which determine the specific route of entry. Virus entry is an active process that can trigger host-cell signaling immediately upon receptor engagement, leading to the activation of pathways such as MAPK/ERK, PI3K-AKT, and Src-family kinases that regulate endocytosis, as well as cytoskeletal remodeling and virus trafficking (49). Therefore, one contributing factor in our results may be differential expression of viral entry receptors at the cell surface. For EHV-1, the cellular receptor repertoire is still not fully elucidated, making it difficult to directly relate early biophysical changes to specific entry pathways (50). In contrast, for EAV, recent studies indicate that the tetraspanin CD81 is a likely receptor or co-receptor, although the level and distribution of CD81 expression in primary equine lung cells remain unknown (51). Variability in receptor abundance or accessibility between primary cells and continuous cell lines could therefore modulate early post-entry responses detectable by DHT. For herpesviruses, tegument proteins delivered upon viral entry are known to rapidly modulate host signaling pathways and reorganize cellular architecture, including the actin cytoskeleton (52). In EHV-1, actin remodeling has been described and has functional implications for the viral replication cycle (53). Because the refractive index measured by DHT reflects the local concentration and spatial distribution of cellular material, early virus-induced changes in cytoskeletal organization, nucleolar structure, and intracellular mass distribution provide a plausible explanation for the RI alterations observed shortly after infection. This may account for the very early RI changes and nucleolar fragmentation detected in EHV-1-infected primary equine lung cells. In contrast, the early RI changes observed during EAV infection, especially in RK-13 cells, are more difficult to interpret. In our experimental system, EAV uncoating was completed by 1 h post-infection. Thus, for this arterivirus, the 0 h time point primarily reflects virus binding, the 1 h time point is expected to capture the changes associated with entry, fusion, and intracellular trafficking, whereas the 2 h time point likely includes very early post-entry events, including the onset of viral translation, consistent with the positive-sense RNA genome of EAV (Fig. S9). However, because nonstructural protein (nsp) expression begins approximately 2 h post-infection, it seems unlikely that the very early RI changes detected in RK-13 cells can be fully explained by nsp activity alone (54). An alternative explanation is that viral structural proteins may exert host-modulatory effects immediately upon entry. In support of this, the major arteriviral envelope protein GP5 has been shown to antagonize innate immune signaling by targeting TRIF-mediated IFN-β induction through TRIF degradation (55). While this does not directly account for the early RI changes observed here, it suggests that incoming viral proteins may influence host cell physiology before substantial nonstructural protein expression occurs. Differences between RK-13 cells and primary equine lung cells in signaling competence and cellular phenotype may therefore contribute to the distinct RI dynamics observed following EAV infection.

Despite both cell types being permissive to all three viruses, ERBV infection did not induce detectable changes in the RI, area, or volume, although rapid alterations were observed using IncuCyte-based analysis at later time points. This discrepancy likely reflects fundamental differences in entry mechanisms. ERBV is a non-enveloped virus, more simple in virion composition, whereas EHV-1 and EAV are enveloped viruses that trigger membrane fusion events associated with entry (56).

Some of the volume and area changes detected in this study were not fully recapitulated by endpoint actin immunofluorescence imaging. This was particularly apparent in EHV-1-infected cells, where morphological differences were observed between the two imaging approaches. A plausible explanation is that holotomographic imaging was performed on live cells without fixation, thereby minimizing artifacts and selection bias associated with sample processing. By contrast, immunofluorescence required washing and fixation steps that may have preferentially displaced more rounded and weakly adherent cells, thus altering the composition of the analyzed cell population. These observations underscore the value of label-free live-cell imaging for detecting early infection-associated morphological changes while reducing bias introduced by endpoint processing. In a representative study, DHT was applied to Vaccinia virus, herpes simplex virus type 1 (HSV-1), and human rhinovirus infections in HeLa cells, revealing virus-specific refractive index gradient (RIG) changes that emerged predominantly at later stages of infection, from ~4 h post-infection for Vaccinia virus to ~8–10 h for HSV-1 and rhinovirus, and were largely dependent on active viral replication (18). In contrast, our work extends these observations by examining veterinary viruses in non-transformed and primary cells and suggests that spatially resolved RI measurements can capture localized intracellular reorganization much earlier, in some cases, as early as 2 h post-infection. Together, these findings indicate that while DHT is increasingly recognized as a powerful tool to study CPE, its application to early infection events and diverse virus-host systems remains underexplored and warrants broader use.

We then focused on analyzing more local changes that could be directly induced by the infection. We defined the most frequently recurring features corresponding to specific RI value ranges, which, after applying pseudocoloring, allowed for their clear visualization, differentiation, and calculations. The RI was measured for selected organelles, namely nucleoli and lipid droplet-like structures. In the present study, these structures were referred to as lipid droplet-like rather than definitively identified as lipid droplets because, although high-RI lipid droplets have been validated previously in label-free holotomographic and optical diffraction tomography studies (57, 58), we did not perform orthogonal validation in our own infection model. This was further limited by the fact that our DHT system does not include a fluorescence imaging module, precluding direct correlative confirmation in the same experimental setup.

In this study, we did not observe statistically significant changes in the area or volume of lipid droplet-like structures. The only detectable effect was an increase in RI in ELCs infected with EHV-1, which may indicate early virus-induced changes in intracellular lipid organization without overt alterations in lipid droplet-like structure size. This interpretation is consistent with previous reports showing that viral infection can rapidly affect cellular lipid composition and organization at early stages, even in the absence of obvious morphological changes in lipid droplets (59, 60). However, interpretation of these results should be made with caution, as some groups of virus-infected cells exhibited cytoplasmic condensation and reduced cell volume, which altered intracellular density and affected the distribution of RI values and contrast in 3D-RI tomograms. These changes, confirmed by increased mean cellular RI and dry mass together with decreased cell volume, made the isolation and segmentation of individual lipid droplet-like structures progressively more difficult as the cytopathic effect advanced. As a consequence, the number of cells in which individual lipid droplet-like structures could be reliably segmented decreased over time after infection, which reduced sample size and may have limited statistical power in the infected groups.

Changes in the nucleoli were also observed in EHV-1-infected ELCs, suggesting that cell type is crucial for the observation of early morphological changes in herpesviruses. While not conclusive, these findings reinforce the need to include both immortalized and primary cell models in viral pathogenesis studies, particularly when investigating viruses with multi-tissue tropism (61).

Analysis of the protrusions and nanotubes showed that the most interesting changes occurred in the primary cell line, where the length of the protrusions during EAV infection showed the strongest trends of change. Despite the limited number of objects in the ELCs line, which made it impossible to obtain statistical significance, these observations indicate a potentially biologically significant effect that requires further analysis on a larger number of cells in the future. The nanotubes or tunneling nanotubes are structures that can form remarkably quickly in response to cellular stimuli, with membrane protrusions appearing within minutes and maturing into functional conduits capable of cytoplasmic exchange within 30 min, driven by actin polymerization and early signaling (62). For bovine herpesvirus, a virus very closely related to EHV-1, these structures were highly variable in size, some extending beyond 20 µm, and were shown to play a crucial role in direct cell-to-cell viral spread (63).

Overall, these results indicate that early RI increases are tightly linked to reductions in cell area and volume and reflect virus- and cell-type-specific biophysical responses. To our knowledge, this is the first time DHT was used to observe the virus entry of viruses from distinct families and in two cell types. Previous limited studies indicated the usefulness of addressing morphological changes in virus-infected cells (18). Recently, the DHT was used in the study of virus egress and the maturation of assembly compartment (64).

Nonetheless, DHT is not without limitations (65). Its application requires specialized instrumentation and technical expertise (66, 67), which are not yet standard in virology laboratories. Our analysis indicates that different viruses leave subtle yet consistent morphological “footprints” in infected cells (68). Although this study was not designed to classify viruses based solely on morphology, EHV-1, EAV, and ERBV induced distinct early spatiotemporal patterns involving nucleoli, lipid droplets, and RI. These findings suggest that early infection stages are associated with characteristic RI-derived signatures that may serve as informative markers of virus-specific cellular remodeling. Such approaches could be further strengthened by machine-learning-based image analysis to enable automated recognition of early virus-induced patterns (69, 70).

Importantly, beyond diagnostics, DHT offers unique advantages for basic research by enabling real-time, label-free visualization of early host-pathogen interactions (71). Given its non-destructive nature (72), DHT may be particularly valuable for monitoring antiviral responses and infection kinetics at single-cell resolution, providing dynamic insights into viral inhibition and clearance processes (22, 73, 74).

A limitation of this study is that the DHT analyses were not performed as a replicate-based biological inference framework. Instead, the data set was generated as an exploratory image-based analysis of individual cells and selected subcellular structures obtained at defined early time points after synchronized infection. Thus, the statistical comparisons should be interpreted as evidence for early quantitative trends within the analyzed data set rather than as definitive effect estimates across independent biological replicates. Further studies, including independent replicate-level designs, will be needed to validate the robustness and generalizability of these observations. Moreover, DHT data were acquired at discrete time points rather than as continuous single-cell recordings, precluding direct analysis of relative changes within the same cell over time. It was related to high migration of single cells in time and their overlapping behavior observed in preliminary experiments (75). Moreover, at this stage, DHT alone did not allow direct identification of infected individual cells in the present study; therefore, the observed readouts should be interpreted as population-level responses under synchronized high-MOI infection conditions. At the same time, the detected early DHT-derived changes suggest that this approach may have future potential for more direct discrimination of infected cells, provided that this is validated in dedicated follow-up studies. Future studies combining DHT with improved long-term single-cell tracking or matched infection-status validation will be important to determine to what extent these early responses arise uniformly across the infected cell population. Furthermore, incorporating longer DHT recordings and improved single-cell tracking will be important to further explore the temporal dynamics of early infection-associated cellular remodeling.

### Conclusion

The study focused on infection-associated changes that were detectable as early as 2 h after infection. To our knowledge, no previous study has reported such an early quantitative characterization in the context of veterinary-relevant viruses. Conventional light microscopy confirmed that the onset of cytopathic changes was consistent with known viral replication kinetics, providing a qualitative reference for subsequent DHT-based analyses.

Obtained results indicate that DHT enables earlier quantitative characterization than conventional light microscopy in this system. While traditional CPE monitoring and automated live-cell imaging (e.g., IncuCyte) rely on late-stage morphological shifts, DHT captures the "biophysical signature" of infection within the first 1–2 h.

By utilizing the RI as a label-free, quantitative biomarker, we successfully visualized virus-specific intracellular remodeling, including nucleolar fragmentation and lipid droplet-like structures reorganization, that remains undetectable by standard light microscopy. Our findings suggest that DHT not only eliminates the need for time-consuming fixation and labeling.

Ultimately, DHT provides a valuable platform for resolving early virus-host dynamics. The identification of distinct RI-based signatures for EAV, EHV-1, and ERBV underscores the potential of this technology to be integrated with machine-learning frameworks, offering a path toward rapid, automated, and non-destructive viral diagnostics and antiviral drug screening. Future integration with machine-learning algorithms could enable the automated recognition of these early “refractive index fingerprints,” potentially creating a rapid, label-free diagnostic platform.

## SUPPLEMENTAL MATERIAL

Figure S1 msphere.00304-26-s0001.tif Automated live cytopathic effect imaging using the IncuCyte system.

Fig. S2 msphere.00304-26-s0002.tif Single-cell distributions of whole-cell parameters in ELCs.

Fig. S3 msphere.00304-26-s0003.tif Baseline-normalized whole-cell parameters in ELCs.

Fig. S4 msphere.00304-26-s0004.tif Single-cell distributions of whole-cell parameters in RK-13 cells.

Fig. S5 msphere.00304-26-s0005.tif Baseline-normalized whole-cell parameters in RK-13 cells.

Fig. S6 msphere.00304-26-s0006.tif Observed trends in tubular intercellular connections in RK-13 and ELCs.

Fig. S7 msphere.00304-26-s0007.tif Cytoskeleton reorganization induced by viral entry events over time.

Fig. S8 msphere.00304-26-s0008.tif Confocal microscopy images of actin remodeling.

Fig. S9 msphere.00304-26-s0009.tif Visualization of EAV particles on the cells of infected cells.

Table S1 msphere.00304-26-s0010.pdf Exact numbers of analyzed cells and subcellular structures included in each DHT analysis.

ASM does not own the copyrights to Supplemental Material that may be linked to, or accessed through, an article. The authors have granted ASM a non-exclusive, world-wide license to publish the Supplemental Material files. Please contact the corresponding author directly for reuse.
