# Supplementary material for: Label-free quantification of early virus-induced cellular responses by digital holographic tomography
Source: mSphere. 2026 Aug 4;11(8):e00304-26. doi: 10.1128/msphere.00304-26 (PMC13508796; doi:10.1128/msphere.00304-26)
Supplement: Supplementary file 1 [file msphere.00304-26-s0010.pdf]

Cell type	Analysis	Condition	Time point	n
**RK-13**	whole cell	Control	0 h	15
**RK-13**	whole cell	Control	2 h	12
**RK-13**	whole cell	EAV	0 h	10
**RK-13**	whole cell	EAV	1 h	10
**RK-13**	whole cell	EAV	2 h	16
**RK-13**	whole cell	EHV	0 h	10
**RK-13**	whole cell	EHV	1 h	15
**RK-13**	whole cell	EHV	2 h	11
**RK-13**	whole cell	ERBV	0 h	15
**RK-13**	whole cell	ERBV	1 h	12
**RK-13**	whole cell	ERBV	2 h	19
**RK-13**	nucleoli	Control	0 h	15
**RK-13**	nucleoli	Control	2 h	12
**RK-13**	nucleoli	EAV	0 h	13
**RK-13**	nucleoli	EAV	1 h	10
**RK-13**	nucleoli	EAV	2 h	13
**RK-13**	nucleoli	EHV	0 h	10
**RK-13**	nucleoli	EHV	1 h	14
**RK-13**	nucleoli	EHV	2 h	12
**RK-13**	nucleoli	ERBV	0 h	15
**RK-13**	nucleoli	ERBV	1 h	14
**RK-13**	nucleoli	ERBV	2 h	13
**RK-13**	lipid droplets	Control	0 h	10
**RK-13**	lipid droplets	Control	2 h	12
**RK-13**	lipid droplets	EAV	0 h	13
**RK-13**	lipid droplets	EAV	1 h	10
**RK-13**	lipid droplets	EAV	2 h	13
**RK-13**	lipid droplets	EHV	0 h	10
**RK-13**	lipid droplets	EHV	1 h	14
**RK-13**	lipid droplets	EHV	2 h	12
**RK-13**	lipid droplets	ERBV	0 h	15
**RK-13**	lipid droplets	ERBV	1 h	14
**RK-13**	lipid droplets	ERBV	2 h	13
**RK-13**	protrusions	EAV	2 h	11
**RK-13**	protrusions	EHV	2 h	8
**RK-13**	protrusions	ERBV	2 h	7
**ELCs**	whole cell	Control	0 h	12
**ELCs**	whole cell	Control	2 h	10
**ELCs**	whole cell	EAV	0 h	20
**ELCs**	whole cell	EAV	1 h	19
**ELCs**	whole cell	EAV	2 h	19
**ELCs**	whole cell	EHV	0 h	14
**ELCs**	whole cell	EHV	1 h	14
**ELCs**	whole cell	EHV	2 h	12
**ELCs**	whole cell	ERBV	0 h	20
**ELCs**	whole cell	ERBV	1 h	17
**ELCs**	whole cell	ERBV	2 h	18
**ELCs**	nucleoli	Control	0 h	18
**ELCs**	nucleoli	Control	2 h	12
**ELCs**	nucleoli	EAV	0 h	29
**ELCs**	nucleoli	EAV	1 h	28

**ELCs**	nucleoli	EAV	2 h	28
**ELCs**	nucleoli	EHV	0 h	19
**ELCs**	nucleoli	EHV	1 h	17
**ELCs**	nucleoli	EHV	2 h	10
**ELCs**	nucleoli	ERBV	0 h	26
**ELCs**	nucleoli	ERBV	1 h	21
**ELCs**	nucleoli	ERBV	2 h	19
**ELCs**	lipid droplets	Control	0 h	15
**ELCs**	lipid droplets	Control	2 h	10
**ELCs**	lipid droplets	EAV	0 h	26
**ELCs**	lipid droplets	EAV	1 h	24
**ELCs**	lipid droplets	EAV	2 h	27
**ELCs**	lipid droplets	EHV	0 h	17
**ELCs**	lipid droplets	EHV	1 h	17
**ELCs**	lipid droplets	EHV	2 h	10
**ELCs**	lipid droplets	ERBV	0 h	26
**ELCs**	lipid droplets	ERBV	1 h	23
**ELCs**	lipid droplets	ERBV	2 h	28
**ELCs**	protrusions	EAV	2 h	10
**ELCs**	protrusions	EHV	2 h	8
**ELCs**	protrusions	ERBV	2 h	10

Table S1. Exact numbers of analyzed cells and subcellular structures included in each DHT analysis.
